# Interpretability of Causal Discovery in Tracking Deterioration in a Highly Dynamic Process

**DOI:** 10.3390/s24123728

**Published:** 2024-06-08

**Authors:** Asha Choudhary, Matej Vuković, Belgin Mutlu, Michael Haslgrübler, Roman Kern

**Affiliations:** 1 Pro2Future GmbH, Inffeldgasse 25F, 8010 Graz, Austria; matej.vukovic@pro2future.at (M.V.); belgin.mutlu@pro2future.at (B.M.); 2 Pro2Future GmbH, Altenberger Straße 69, 4040 Linz, Austria; michael.haslgruebler@pro2future.at; 3 Institute of Interactive Systems and Data Science (ISDS), Graz University of Technology, Rechbauerstraße 12, 8010 Graz, Austria; rkern@tugraz.at

**Keywords:** degradation monitoring, health monitoring, causal discovery, jaccard distance, interpretability, causal interpretability

## Abstract

In a dynamic production processes, mechanical degradation poses a significant challenge, impacting product quality and process efficiency. This paper explores a novel approach for monitoring degradation in the context of viscose fiber production, a highly dynamic manufacturing process. Using causal discovery techniques, our method allows domain experts to incorporate background knowledge into the creation of causal graphs. Further, it enhances the interpretability and increases the ability to identify potential problems via changes in causal relations over time. The case study employs a comprehensive analysis of the viscose fiber production process within a prominent textile industry, emphasizing the advantages of causal discovery for monitoring degradation. The results are compared with state-of-the-art methods, which are not considered to be interpretable, specifically LSTM-based autoencoder, UnSupervised Anomaly Detection on Multivariate Time Series (USAD), and Deep Transformer Networks for Anomaly Detection in Multivariate Time Series Data (TranAD), showcasing the alignment and validation of our approach. This paper provides valuable information on degradation monitoring strategies, demonstrating the efficacy of causal discovery in dynamic manufacturing environments. The findings contribute to the evolving landscape of process optimization and quality control.

## 1. Introduction

Detecting degradation in industrial processes is a critical undertaking that requires quick and precise actions. Degradation, whether subtle or pronounced, serves as an early warning of potential issues within the system, indicating factors like equipment wear, material variations, or environmental changes. Timely detection and increased understanding are paramount to avoid larger problems, ensuring operational efficiency and mitigating unexpected failures. In response to identified degradation, a proactive strategy that integrates advanced monitoring technologies and data analytics allows for precise interventions, including scheduled inspections, parameter adjustments, or component replacements as part of predictive maintenance initiatives. This strategic approach not only reduces the risk of unforeseen failures, but also optimizes maintenance practices by addressing issues precisely when necessary.

To ensure that the quality and quantity of the final product fulfills given criteria, industries frequently deploy preventive measures. These measures rely on distinct maintenance strategies, and are categorized into breakdown maintenance, planned maintenance, and condition-based maintenance or predictive maintenance as discussed in [1], to address equipment reliability and performance effectively.

Despite the importance of degradation monitoring for maintaining product quality, many industries still rely on reactive maintenance, which can result in costly downtime and losses. Proactive strategies for early detection of degradation are crucial for preventing larger issues and enhancing customer satisfaction. Therefore, there is a very high need for innovative degradation monitoring methods to ensure continuous operation and success in industrial processes.

Degradation monitoring involves the systematic observation and analysis of changes in the performance or quality of a system or process over time. It aims to identify deviations from normal operating conditions that may indicate deterioration or wear in equipment, infrastructure, or production processes. By continuously monitoring key indicators or variables, degradation monitoring allows for early detection of potential issues, enabling proactive maintenance or intervention to prevent failures, optimize performance, and ensure the longevity and reliability of assets and operations [2]. Extensive research has been conducted on monitoring degradation within industrial processes, aimed at detecting and addressing faulty components early to prevent any compromise in product quality. These studies are extensively reviewed in Section 2, starting with insights on causal discovery and the techniques applicable for identically distributed (i.i.d.) data and time series data and then further exploring the ongoing efforts aimed at devising effective strategies for maintaining process integrity and product consistency.

Our approach to monitoring degradation uses causal discovery, leveraging the inherent cause-and-effect relationships in degradation. Causal discovery is the process of identifying cause-and-effect relationships among variables in a dataset or system. It involves determining how changes in one variable influence changes in another and the direction of these effects [3]. This means that degradation in one sensor or component can lead to degradation in others or in the output variable. Additionally, using causality analysis for degradation monitoring allows domain experts to incorporate their process knowledge, enriching the understanding of the system. We utilize the Fast Causal Inference (FCI) algorithm, which was initially crafted for independent and identically distributed (i.i.d.) data, but has been tailored by us to accommodate temporal dependencies inherent in time series data. FCI is chosen because it can effectively conduct causal discovery even in scenarios with latent confounders [3], which is a common occurrence in real-world situations where not all parameters are directly measured.

An essential aspect of our approach involves selecting a reference causal graph that embodies the ideal or normal working condition. We then track degradation by comparing subsequent causal graphs over time against this reference, utilizing the Jaccard distance as a metric of dissimilarity. In our case study (refer to Section 4), where machine components are replaced every 6 months, the reference causal graph is chosen close to the timeframe when the components are new. Subsequent causal graphs are compared against it, and the Jaccard distance is calculated, followed by trend analysis. When the Jaccard distance exceeds a predefined threshold set by domain experts, they can investigate the cause-and-effect relationship of this deviation using the corresponding causal graphs.

After conducting degradation monitoring, it is crucial that the results are interpretable to domain experts, enabling them to take necessary actions. Our approach is based on causal discovery to address this challenge by providing interpretable causal graphs, allowing domain experts to discern changes in the process. This focus on transparency aligns with the principles of explainable AI, which aims to elucidate the reasoning behind model decisions and outputs [4]. By providing clear insights into causal relationships, our approach facilitates informed decision-making and fosters trust in the monitoring system’s results. Additionally, to interpret the results obtained from causal graphs, we have developed a visualization illustrating dynamic changes in features over time. This visualization depicts the types of relationships between feature pairs over time and highlights the presence of new connections or confounders in the current causal graph compared to previous ones. These new relations indicate changes in the underlying process that may represent critical deviations in the manufacturing process itself. In summary, our contributions in the context of the viscose fiber production process include the following:

- Proposing a novel approach making use of causal discovery and adapting the FCI algorithm to time series data for tracking degradation processes together with proposed distance measures to quantify these changes;
- Developing visualizations to illustrate dynamic changes as a tool for communication with domain experts achieving the goal of interpretable results;
- Comparing our degradation monitoring results with those obtained using the state-of-the-art LSTM-based autoencoder, Deep Transformer Networks for Anomaly Detection in Multivariate Time Series Data (TranAD), and UnSupervised Anomaly Detection on Multivariate Time Series (USAD) methods, which are not considered to be interpretable.

In response to this need, our work focuses on monitoring degradation in a dynamic production process, particularly in the realm of viscose fiber production, as elaborated in Section 4. Our approach centers on leveraging the causal discovery technique FCI (Fast Causal Inference). Employing causal discovery for degradation monitoring offers a two-fold advantage. Firstly, it enables domain experts to integrate background knowledge into the creation of causal graphs. Secondly, it facilitates the examination of changes in causal relations at specific time points, enhancing interpretability and paving the way for deeper investigations, including root cause analysis and counterfactual reasoning.

Our contribution encompasses a comprehensive approach, beginning with the selection of the most suitable algorithm designed to the specific requirements of the problem at hand, which is to monitor degradation in a continuous and highly dynamic production process. We then adapt the chosen algorithm to accommodate time series data, recognizing that neglecting temporal information can obscure the dependencies between subprocesses and their temporal evolution.

Incorporating domain knowledge about the process is a crucial aspect of our approach. We acknowledge the fundamental principle that past events cannot be influenced by present or future events, which may seem intuitive in a process context but is not always evident when analyzing historical data. This integration of domain knowledge enhances the robustness and interpretability of our methodology.

Another key element of our approach is the method to select a reference graph that accurately represents the ideal working condition of the system. This reference graph serves as a benchmark for comparison, allowing us to identify deviations and quantify degradation effectively in the process using a prominent measure (Jaccard distance).

Finally, we assess the efficacy of our approach by comparing the degradation detected using the Jaccard distance measure with that of a state-of-the-art methods, specifically with LSTM-based Autoencoder (AE), Deep Transformer Networks for Anomaly Detection in Multivariate Time Series Data (TranAD) and UnSupervised Anomaly Detection on Multivariate Time Series (USAD) methods. This comparative analysis provides valuable information on the efficacy of our methodology in detecting and monitoring degradation within the production process.

Ultimately, our approach empowers domain experts with the means to monitor changes within their process effectively with the help of causal graphs. By providing a framework to track variations and understand their causes, our methodology enables experts to make informed decisions that are well-aligned with the dynamics of the system.

Section 2 provides a background on causal discovery and an overview of existing research on degradation monitoring in the manufacturing industry. Section 3 delves into the methodology employed for monitoring degradation in the production process, providing detailed insights into preliminaries such as data preprocessing and various algorithms used for this purpose. Subsequently, Section 4 presents the results of the methodology applied to the viscose fiber production process within a prominent textile industry. In Section 5, we conduct an evaluation by comparing our results with state-of-the-art methods, specifically Long Short-Term Memory (LSTM)-based Autoencoder (AE), Deep Transformer Networks for Anomaly Detection in Multivariate Time Series Data (TranAD), and UnSupervised Anomaly Detection on Multivariate Time Series (USAD) for monitoring degradation in the process outlined in Section 4. This section not only presents the evaluation but also discusses the findings of the results in comparison to our approach. This paper concludes with Section 6, discussing the key findings and identifying potential avenues for future research.

## 2. Background and Related Work

This section provides an overview of causal discovery and its techniques applicable to identically distributed (i.i.d.) data and time series data. It also explores various approaches found in the literature for monitoring degradation, beginning with a focus on unsupervised learning methods due to the absence of labeled data in real-world industrial settings, as is the case in our case study. The discussion transitions into exploring causal discovery techniques specifically tailored to the manufacturing industry, emphasizing the necessity for interpretability. Following this, a brief overview of Explainable AI (XAI) and its popular techniques is provided. Lastly, the section underscores the importance of causal interpretability in monitoring degradation, as it offers a profound insight into the changes within a highly dynamic causal process.

### 2.1. Causal Discovery

Causal Discovery (CD) refers to the process of identifying and understanding the causal relationships between variables in a system. The objective is to uncover the cause-and-effect relationships that exist between different factors or variables within a dataset or a real-world system [5]. In causal discovery, the emphasis is on *inferring causal structures rather than merely identifying associations or correlations*. It aims to answer questions such as “What causes what?” or “How do changes in one variable influence another?” The ultimate goal is to reveal the underlying mechanisms that govern the observed phenomena [6].

Based on the type of the data, CD algorithms can be divided into two categories [7]:

- CD algorithms for independent and and identically distributed (i.i.d.) data i.e., non-time series data;
- CD algorithms for time series data.

**Causal Discovery algorithms for i.i.d. data.** For a dataset to be i.i.d. the following rules must be met [8]:

- Independent: Each observation is not influenced by or dependent on any other observation. The occurrence or value of one data point does not affect the occurrence or value of another.
- Identically Distributed: All observations come from the same probability distribution. This implies that underlying statistical properties, such as mean, variance, and other distributional characteristics, do not change.

These are non-time series data, as time series data points are typically not independent because each observation in a time series is often influenced by and dependent on previous observations. The value of a data point at a given time is often related to its past values. Also, time series data often exhibit temporal patterns, trends, and seasonality, making the observations not identically distributed across time. The statistical properties of data points may change over time due to external factors or underlying dynamics [7].

Causal discovery for i.i.d. data relies on statistical and computational methods to infer causal relationships among variables. Among the most popular algorithms in this category are the constraint-based causal discovery algorithms such as the Peter and Clark (PC) algorithm and the Fast Causal Inference (FCI) algorithm. These algorithms identify causal structures by systematically testing conditional independence relationships in the data [7]. However, Fast Causal Inference (FCI) is regarded an enhancement over the Peter and Clark (PC) algorithm in the context of causal discovery tasks. The primary advantage of FCI lies in its ability to effectively handle latent (unobserved) variables or confounders. A confounder is a variable that is correlated with both the cause and the effect, potentially introducing a misleading association between them and distorting the true relationship [9]. Latent variables or confounders can introduce confounding in causal relationships, therefore the FCI algorithm is used for causal discovery as it incorporates techniques to address these confounders, making it more robust in the presence of unobserved variables [3]. The functioning of both PC and FCI can be briefly explained as follows [7]:

- Skeleton Construction: The PC algorithm begins by constructing an undirected graph, called the skeleton, based on conditional independence tests.
- Conditional Independence Tests: It tests for conditional independence between variables to identify potential causal relationships.
- V-Structure Identification: It identifies V-structures, which are indicative of potential causal relationships, in the undirected graph.
- Edge Orientation: The PC algorithm orients edges in the graph to form a partially directed acyclic graph (PDAG) by exploiting the identified V-structures.

To address latent confounders, FCI requires a substantially higher number of conditional independence tests compared to PC. In the worst-case scenario, this requirement escalates exponentially with the number of features present in the data [7].

**Causal Discovery algorithm for time series data:** Among the most popular causal discovery algorithms for time series data are the tsFCI and PCMCI algorithm. The time series Fast Causal Inference (tsFCI) algorithm, adapted from the Fast Causal Inference (FCI) algorithm for non-temporal variables, is designed to infer causal relationships from time series data. It operates in two distinct phases: (i) an adjacency phase and (ii) an orientation phase. Leveraging temporal priority and consistency across time, it employs these phases to orient edges and constrain conditioning sets. The tsFCI algorithm yields a window causal graph, offering the advantage of detecting lagged hidden confounders. However, it comes with limitations, as it is unable to model cyclic contemporaneous causation and instantaneous relationships [7]. However, in the viscose fiber production process described in Section 4, the process consists of a cyclic behavior that involves two phases, namely the rejection and the filtration phases. Also, as described in Section 4.2, the data are a multivariate time series, thus having an instantaneous relationship between the features/variables. Due to these limitations of the tsFCI algorithm, it was not employed in our analysis.

Next is the PCMCI algorithm, which is designed for large-scale time series data, addressing challenges encountered when adding more variables. In such datasets, there is a risk of reduced power in causal analysis, potentially resulting in overlooked original causal relationships. PCMCI addresses this issue by employing a two-stage approach. First, it selects relevant conditions using a variant of the PC algorithm, known as PC1, to remove irrelevant variables. Then, in the second stage, it utilizes the momentary conditional independence (MCI) test to mitigate false positive rates, even in highly correlated data. Whether two variables are independent given their parent sets is evaluated in the second stage, i.e., by the MCI test. This is mathematically formulated as follows [7]:

$$X_{t-m}^{a}⫫X_{t}^{b}|PA\left(X_{t-m}^{a}\right),PA\left(X_{t}^{b}\right)$$

where $X_{t-m}^{a}$ is variable $X^{a}$ at time instant t−m, $X_{t}^{b}$ is variable $X^{b}$ at time instant *t*, and $PA(X_{t-m}^{a})$ and $PA(X_{t}^{b})$ are their parents, respectively [7]. PCMCI assumes stationarity, time-lagged dependencies, and causal sufficiency in the data. It typically outperforms the PC algorithm even when stationarity is violated. However, it is not suitable for highly predictable systems with minimal new information at each time step. In real-world datasets, variables often exhibit varying degrees of influence, with some exerting more impact than others. Given that degradation typically manifests as a gradual change rather than a sudden one, the differences between variables may not be substantial, resulting in minimal new information at each time step. Consequently, PCMCI may not be the optimal choice for our dataset. As a result, we did not employ PCMCI in our case study. Instead, the method we employed is detailed in Section 3.2.

### 2.2. Approaches to Unsupervised Degradation Monitoring

This section delves into various methodologies employed in the literature for unsupervised degradation monitoring, fault detection, predictive maintenance, condition monitoring, and machine and sensor health monitoring. These approaches aim to identify deterioration in processes without relying on predefined ground truth data, i.e., in an unsupervised manner.

One of the widely adopted methods for unsupervised anomaly detection is the One-class Support Vector Machine (SVM), as discussed in [10]. It involves training on normal data and then identifying anomalies in test data. Modifications like Robust One-class SVM and eta One-class SVM aim to improve its robustness, especially against outliers, with promising results shown by the eta One-Class SVM. In [11], a kernel-based SVM monitors sensor data to assess the health status of a complex industrial asset—an auxiliary marine diesel engine. Additionally, ref. [12] applies One-class SVM for fault detection in a closed-loop feedback controller system. Traditional One-class SVMs are designed for i.i.d. data, which do not encompass typical time series data. To address this, ref. [13,14] convert time series data into vectors to enable One-class SVM application.

Autoencoders (AE) and Long Short-Term Memory (LSTM) networks are widely used for anomaly detection. Studies like [15,16,17] utilize AE for this purpose. In [18], deep AE is applied to monitor equipment health condition, using reconstruction error as a key health indicator. Their method was tested on public datasets FD001, FD003, and Mill, comparing favorably with state-of-the-art approaches. Another study by [19] focuses on degradation detection in machine bearings. They employ a sparse autoencoder to extract unsupervised features and calculate the Autoencoder Correlation (AEC)-based rate between initial and successive samples. This rate effectively identifies the onset of degradation in machine components.

The review in [20] explores ARIMA (Autoregressive Integrated Moving Average), ARIMAX (Autoregressive Integrated Moving Average Exogenous), and VAR (Vector Autoregression) as deep learning models for anomaly detection. It addresses ARIMA’s limitation in handling multivariate time series data by introducing the ARIMAX model, including an additional explanatory variable, or using VAR, which utilizes vectors to accommodate multivariate terms.

In [21], an LSTM-based autoencoder (AE) is employed for anomaly detection in time series data of customer demand in supply chain management. The method trains the model on normal data and utilizes it to predict future steps in the time series, quantifying discrepancies between predicted and observed values as prediction errors. A kernel estimator of the quantile function establishes a threshold for anomaly detection, achieving a lower false alarm rate compared to traditional One-class Support Vector Machine methods. A similar concept as that of [21] is applied in our evaluation, as detailed in Section 5. Online anomaly detection using LSTM-based AE on multivariate time series data is explored for Smart Manufacturing in [22]. Additionally, ref [23] addresses anomaly detection and prevention in modern manufacturing processes by leveraging sensor data. The study focuses on scenarios with distributed time series measurements, employing Vector Autoregressive (VAR) modeling for multivariate time series analysis.

In [24], the challenge of monitoring ongoing degradation in lithium-ion battery production lots is addressed by employing five data-driven methods: regression model with prediction bounds, one-class support vector machine, local outlier factor, Mahalanobis distance, and sequential probability ratio test. Since no single method consistently provides the earliest warning of degradation, the authors propose an ensemble approach. This methodology offers valuable insights for device companies, aiding in warranty, recall, and technical decision-making based on anomalous degradation behavior detected in ongoing reliability testing of battery samples from production lots. Similarly, ref. [25] reviews various state-of-the-art unsupervised anomaly detection techniques for time series data.

While classical algorithms like One-class SVM and eta One-class SVM are well-known for degradation/anomaly detection, they are not suitable for time series data. Among traditional unsupervised learning methods, options like AE, LSTM-based AE, ARIMA, ARIMAX, and VAR exist for time series analysis, but ARIMA is not applicable to multivariate time series like ours. Hence, we opt for the LSTM-based AE to evaluate our approach’s effectiveness in degradation monitoring for continuous processes.

Traditional process monitoring methods struggle to adapt to dynamic industrial environments, prompting the development of more flexible approaches. In [26], authors propose a novel method called Element-aware Lifelong Dictionary Learning (EaLDL) to address this challenge. Further, the authors of [27] introduce Jointly Mode-matching and Similarity-preserving Dictionary Learning (JMSDL) to address the challenge of adapting process monitoring models to new modes in industrial processes. JMSDL updates the model using new mode data while preserving representation ability for historical data through a similarity metric.

In addition to the methods discussed earlier, there are several deep learning-based approaches for anomaly detection in time series data, as detailed in [28]. Among these approaches are TranAD and USAD, highlighted in [28]. TranAD, or Deep Transformer Networks for Anomaly Detection in Multivariate Time Series Data [29], utilizes an attention-based mechanism inherent to transformers. The model employs a two-phase adversarial training approach to ensure robust generalization for anomaly detection in arbitrary data sequences. During the first phase, TranAD reconstructs input sequences, and in the second phase, it utilizes the reconstruction error, termed the focus score, to extract short temporal trends, referred to as self-conditioned outputs, from regions exhibiting high deviations.

TranAD has been evaluated on six publicly available datasets, demonstrating superior performance compared to traditional anomaly detection methods. Additionally, it has been applied in [30] to analyze stock market data by comparing it with its predicted version, enabling the detection of deviations from normal price data. The study in [30] concludes that TranAD is highly effective in accurately detecting anomalies. Furthermore, a review paper [31] suggests that anomaly detection using transformer models, such as TranAD, surpasses conventional methods in performance. Hence, the other method that is used to evaluate our approach is the TranAD.

USAD, which stands for UnSupervised Anomaly Detection on Multivariate Time Series [32], is an anomaly detection algorithm designed for multivariate time series data. It leverages adversarially trained autoencoders to address the limitations of traditional autoencoder-based anomaly detection methods. USAD trains a model capable of identifying instances where input data does not contain anomalies, allowing for accurate reconstructions of normal data. Additionally, the autoencoder architecture in USAD enhances stability during adversarial training, mitigating issues such as collapse and nonconvergence observed in Generative Adversarial Networks (GANs). The performance of USAD in detecting anomalies in multivariate time series data has been evaluated on five publicly available datasets [32]. USAD has been recognized for its fast learning capabilities and stability [33].

Ref. [34] offers a comprehensive comparison of various state-of-the-art Deep Anomaly Detection methods, categorizing them based on architecture type and identifying the best performing method for each architecture. Given USAD’s attributes as a fast and stable unsupervised anomaly detection algorithm for multivariate time series data [32], we chose to incorporate it as an additional method to evaluate our approach. Our method offers an additional advantage over traditional approaches in the literature: interpretability for domain experts. This interpretability enables further diagnosis to pinpoint the root cause of degradation in the process, as illustrated by the visualization in Section 4.3.

### 2.3. Causal Discovery in Manufacturing Industry

This section explores the application of causal discovery within the manufacturing sector, focusing on fault diagnosis, root cause analysis, quality problem resolution, predictive maintenance, condition monitoring, and anomaly detection. It outlines the diverse range of applications where causal discovery techniques are utilized to improve operational efficiency and product quality. Continuous research efforts are dedicated to enhancing data-driven algorithms for causal discovery, as they have the potential to identify influential parameters in a given context. In this regard, a recent study conducted by [35] contributes to advancing these algorithms. Here, the authors propose Multi-Scale Neural Network for Granger Causality Discovery (MSNGC), which is a novel approach for analyzing multivariate time series data to discover causal relationships. Unlike existing methods, MSNGC does not need explicit data segmentation between series and time lags. Instead, it extracts causal information across different delay ranges and integrates them using learned attention weights. This comprehensive approach leads to accurate estimation of weighted adjacency matrices, addressing the challenge of discovering causal relationships in time series data.

Ref. [36] proposes a novel data-driven method combining Interpretable Machine Learning (IML) and Process Mining (PM) techniques to construct dynamic causal models for complex industrial processes. Addressing challenges like capturing temporal relations and considering overall performance deterioration, the approach integrates IML and PM to automatically generate causal models. Demonstrated using industrial data from a pulp and paper mill, the method shows promise for enhancing efficiency and control in industrial processes.

In [37], a novel method called the Causality-Gated Time Series Transformer (CGTST) is introduced for diagnosing faults in chemical production processes. It tackles challenges like nonlinearity, nonstationarity, and various forms of noise commonly encountered in chemical process data. CGTST utilizes a Transformer-based model to predict time series variables, assessing causal relationships through a specialized causality gate structure. The method employs causal validation and noise reduction techniques to enhance robustness. Through case studies, CGTST demonstrates superior performance compared to traditional causal discovery approaches, showcasing its potential for industrial fault diagnosis in chemical processes. Validation is conducted on three public datasets: a continuous stirred-tank reactor, the Tennessee Eastman process, and a real-world continuous catalytic reforming process. To address the challenge of fault diagnosis in complex Cyber–Physical Production Systems (CPPSs), [38] proposes a causality-driven hybrid model represented in a Causal Knowledge Graph (CKG). The CKG acts as a transparent system model for collaborative human–machine fault diagnosis in CPPS, offering a solution to unplanned downtimes. The paper introduces a concept for continuous hybrid learning of the CKG, a maturity model to assess fault diagnosis capabilities, and illustrates the industrial setting in the telescopic die production line motivating the approach.

The study in [39] presents a data-driven framework for root cause analysis in Quality Problem Solving (QPS). This framework utilizes extensive QPS data to uncover large-scale causal relationships between quality problems and production factors. A key component is the creation of a Causal Knowledge Graph for Quality Problems (QPCKG), which represents these causal relationships. The process involves classifying QPS data, extracting cause-and-effect slots using causal linguistic patterns, and employing Bidirectional Long-Short-term Memory with Conditional Random Field (BiLSTM-CRF) for core content extraction. A vertex fusion method integrates discrete causalities into the QPCKG. Validated in a real-world application at BBA, a luxury automotive manufacturer, the QPCKG facilitates quality diagnosis and prediction. It provides insights into the fundamental interaction mechanisms between product quality and production factors, aiding decision-making in Root Cause Analysis (RCA). In discrete manufacturing quality problem solving, Ref. [40] proposes a two-stage approach to tackle the complexities of causal relationships. In the first stage, an improved Bayesian network is used to pinpoint likely root causes directly influencing quality indicators. The second stage involves causal inference to estimate the impact of these root causes on the quality indicator. This method enhances the accuracy of root cause identification and allows for quantitative tuning of solutions. The effectiveness of the approach is demonstrated through a case study in aerospace shell part spinning, showcasing precise root cause identification and determination of intervention degree.

To further delve into the field of causal discovery in manufacturing and condition monitoring, a comprehensive review can be found in [41,42].

The objectives of the aforementioned research revolve mainly around anomaly detection, fault diagnosis, condition monitoring, or predictive maintenance. Notably, none of them specifically targets the monitoring of degradation in the process using causal discovery. Although some of these studies share a common initial step of performing causal discovery, their ultimate goals diverge, leading to differences from our approach.

### 2.4. Interpreting Complex Systems: Explainable AI vs. Causal Interpretability

In modern industries, the surge in data availability has prompted a widespread adoption of data-driven modeling approaches, such as machine learning (ML), aimed at enhancing operational efficiency and productivity [43]. While ML models have shown promise in improving performance, their increased complexity often comes at the cost of interpretability, posing challenges, especially in mission-critical scenarios [44]. To address this, Explainable AI (XAI) has emerged as a research focus, aiming to shed light on the decision-making process of complex ML models [45]. XAI refers to the set of techniques and methods used to make Artificial Intelligence (AI) models and their decisions understandable and interpretable to humans. It addresses the “black box” nature of many AI models, where their internal workings are complex and not easily understandable by humans. XAI aims to provide insights into how AI models arrive at their decisions, helping users understand and trust the output of these models [4,46].

In the realm of XAI, two prominent categories of models are intrinsic and post hoc models. Intrinsic models, also known as model-based interpretability, focus on ensuring the interpretability of the model itself by modifying its structure or components. Post hoc models, on the other hand, offer explanations for pre-trained models by scrutinizing both the original model and an additional one, providing insights into their decision-making process [47]. There are several different XAI techniques. Among them the recent XAI methods are Gradient-weighted Class Activation Mapping (Grad-CAM), Local Interpretable Model-agnostic Explanations (LIME), SHapley Additive exPlanations (SHAP), and Trainable attention. All these methods offer post hoc explanations of why a model produced a specific output [47].

In contrast to XAI, which predominantly deals with feature importance and model behavior, causality analysis offers a deeper understanding of complex systems by uncovering cause-and-effect relationships [44]. By integrating domain expertise and tracking process changes over time, causality analysis enables swift anomaly detection and provides actionable insights for system optimization. While XAI methods like SHAP and LIME identify influential features, causality analysis goes beyond by exploring causal relationships, empowering proactive intervention to address root causes and prevent future occurrences [48].

Moreover, causality analysis excels in identifying root causes of events, as demonstrated in the interpretability stage outlined in Section 4.3. By uncovering the underlying mechanisms driving system behavior, it not only aids in diagnosing issues but also empowers proactive intervention to address root causes and prevent future occurrences. This depth of insight distinguishes causality analysis as a powerful tool for degradation monitoring and process optimization.

A recent study [44] provides a good overview of the current state-of-the-art causal interpretability. It provides a classification of existing work into four main categories, that is, causal inference and model-based interpretation, example-based interpretation, fairness, and guarantee of interoperability. The approaches [49,50,51,52] focus on explaining the causal role of different components of the deep neural network in establishing final predictions by calculating average causal effects or establishing a surrogate structural causal model.

Counterfactual explanations aim to find the smallest changes to input data that lead to a model prediction change to a predefined output, aligning well with human reasoning [48]. Multiple works use approaches based on distance measures to generate counterfactual explanations, minimizing errors between model predictions and counterfactual outcomes [53,54]. Extensions like change constraints and adversarial examples have been proposed to enhance the feasibility of counterfactual explanations [55,56]. Recent surveys emphasize utilizing causal graphs for knowledge extraction and process improvement [41,57]. This underscores the need for approaches like ours, which employ causality to establish interpretable causal graphs for monitoring continuous industrial processes.

## 3. Approach

**Figure 1:**
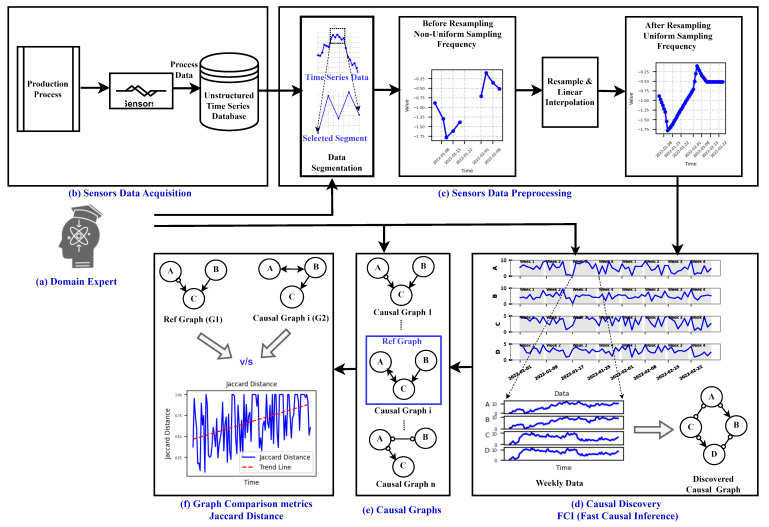
Schematic description of the complete approach, where *A*, *B*, *C* and *D* are nodes representing features/variables of the process.

This section describes the methodology used to monitor degradation in a dynamic production process, providing key foundations for a comprehensive understanding of the approach. The schematic overview of the approach used for degradation monitoring is illustrated in Figure 1. The schematic comprises seven stages denoted Stages a–g.

In Stage a, domain expertise is incorporated, leveraging knowledge for subsequent analyses such as preprocessing of sensor data, causal discovery, creation of causal graphs, and selection of the reference graph. Additionally, domain experts utilize the output of the approach in the interpretability stage (Stage g) for further analysis or visual inspection of the process. The Sensor Data Acquisition stage (Stage b) involves collecting raw data from sensors and other relevant sources to capture the operational dynamics of the production process. The acquired data, typically in the form of unstructured time series data, are then passed to the subsequent stage, Sensor Data Preprocessing (Stage c). In this preprocessing stage, guided by domain knowledge, the data undergo segmentation and resampling.

Subsequently, the process advances to the Causal Discovery stage (Stage d), employing the FCI (Fast Causal Inference) algorithm for causal graph generation. Prior to causal discovery, domain knowledge is leveraged to gather information about the process state (old or new state). The analysis begins from the fresh state of the process, discarding older data.

The FCI algorithm is applied, generating causal graphs for each week (as shown in Stage e). The creation of these causal graphs incorporates domain knowledge, specifically the principle that present or future events cannot influence past events. Next, a reference graph is needed, either from a domain expert or selected from weekly causal graphs based on expert knowledge, which represents the ideal operating condition/state of the process. This reference graph is then compared to all other weekly causal graphs using Jaccard distance metrics, considering the types of edges between each feature pair. This comparison mechanism is depicted in the Graph Comparison metrics stage (Stage f) of the schematic description shown in Figure 1.

Following the computation of Jaccard distance to quantify the dissimilarity between the causal graphs and the reference graph, additional insights such as changes in the causal graph over time can be gleaned through visual examination of these graphs as shown in Figure 1 (Stage g).

### 3.1. Data Preprocessing

As illustrated in Figure 1b, this section outlines the method used to preprocess the raw time series data acquired from the sensors, as depicted in Figure 1b. Given that the production process typically comprises multiple phases, such as filtering of good particles and waste particle removal from the fiber, the data are segmented accordingly based on temporal information pertaining to each phase. Following segmentation, the data undergo resampling and interpolation to ensure uniform sampling frequency. This preprocessing step enhances data consistency and enables smoother subsequent analyses and interpretation. Prior to this preprocessing step, the data may exhibit non-uniform sampling intervals due to factors like shutdowns and maintenance activities, affecting data frequency.

### 3.2. Adapting the Causal Discovery Method (FCI)

Recognizing the constraints of PC, FCI, tsFCI, and PCMCI, we made an adaptation to utilize the FCI algorithm for time series data in our analysis. Typically designed for i.i.d. data, FCI needed modification to accommodate the time-dependent characteristics of our dataset. This adaptation involved augmenting the features with lag values for each feature, enabling FCI to effectively handle the temporal nature of the data. In this context, a lag signifies the time delay between consecutive observations, providing insight into the temporal relationship between a variable and its past values. This approach allowed for the incorporation of additional temporal information, enhancing the applicability of the FCI algorithm to time series data in our analysis.

In summary, the adapted version of FCI follows the following steps:

- Initial Setup: Begin with a set of variables or characteristics. This is given as data = [X,Y,Z,A,B], where *X*, *Y*, *Z*, *A*, and *B* are the column vectors representing the variables or features in the data.
- Data Modification: Modify the data to include lagged versions of the features to capture temporal dependencies. This is given as data=[X_lag0,Y_lag0,Z_lag0,A_lag0,B_lag0,…,X_lag40,Y_lag40,Z_lag40,A_lag40,B_lag40], representing data with lagged versions of the original features up to 40 lags as additional features.
- Graph Formation: Create a complete undirected graph using the variables as vertices.
- Iterative Process: Test pairs of variables for conditional independence given subsets of other variables. Remove edges between variables that are conditionally independent.
- Graph Orientation: Orient edges based on certain criteria, such as the absence of direct causal influence between certain pairs of variables.
- Edge Removal: Further refine the graph by removing edges between pairs of variables that are d-separated given subsets of other variables.

### 3.3. Similarity Measures

Similarity, in the context of causal graphs, refers to the degree of closeness or agreement between two compared entities [58]. In the field of causal discovery, similarity measures serve as quantitative metrics to evaluate the resemblance of structures between different causal graphs, indicating the level of agreement in identified causal relationships. Various similarity measures, such as the Jaccard similarity score, the Sorensen index [59], the Structural Hamming distance, and the Structural Intervention distance [60], are discussed in the literature [58].

**Figure 2:**
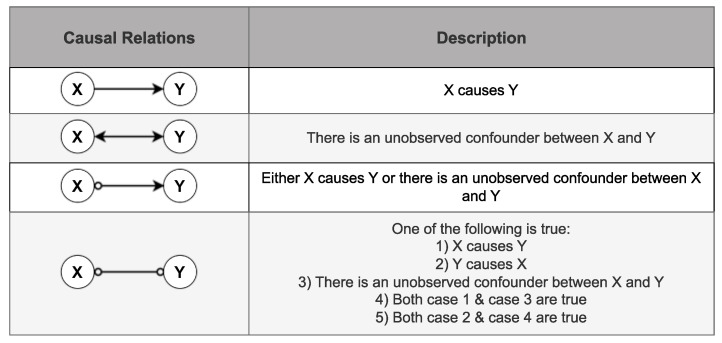
Overview of edge types of FCI based on [61], where *X* and *Y* are two nodes representing features/variables of the process.

Given that the FCI algorithm produces various types of edges, as depicted in Figure 2, the Jaccard similarity score is chosen as a metric. This score measures the similarity of the sets of edges or connections between two graphs by calculating their intersection in relation to the total number of edges [59]. Since the Jaccard similarity score quantifies the degree of similarity between two graphs, a score of 0 indicates complete dissimilarity, while a score of 1 signifies that the two graphs are identical. The Jaccard similarity score is used to determine the reference graph needed to represent the dynamics of the process during ideal/normal operating conditions. However, in the context of degradation monitoring in the viscose fiber production process, described in Section 4, the Jaccard distance, a measure of dissimilarity, is used instead of the Jaccard similarity. This decision is motivated by the need to assess how much the dynamics of the production process have changed since the initial state when all components, including sensors and motors, were in fresh condition. Complementing the Jaccard similarity score, a Jaccard distance of 0 signifies identical graphs, while a value of 1 indicates significant dissimilarity between the graphs.

**Figure 3:**
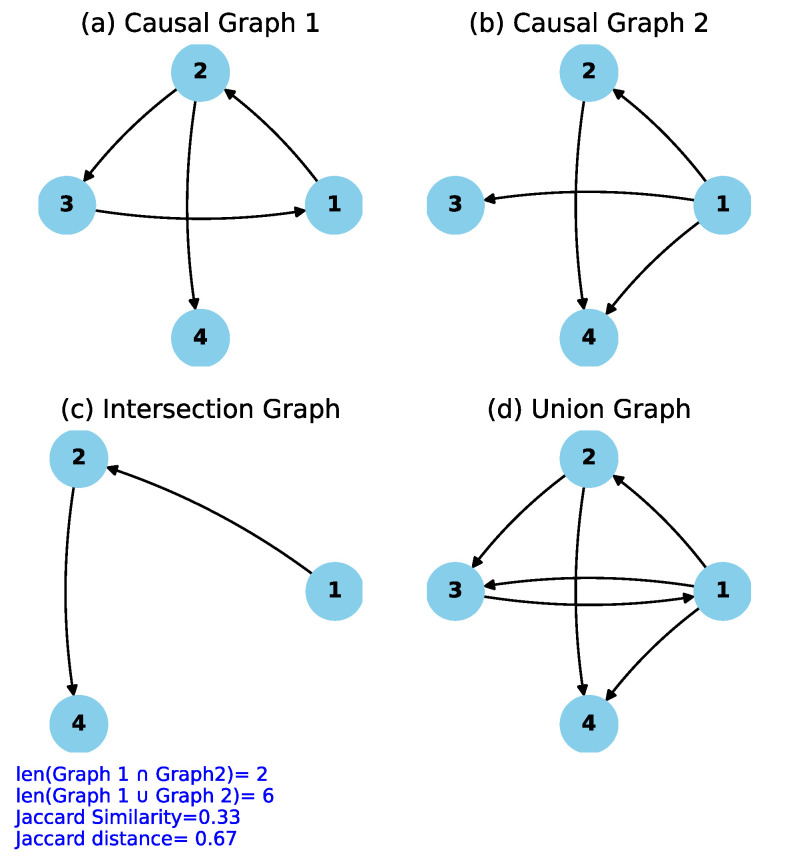
Working of Jaccard similarity score and Jaccard distance. (**a**,**b**) are the two causal graphs to be compared, (**c**,**d**) are the intersection and union of the two graphs (**a**,**b**) [62].

Algorithm 1 depicts the pseudo-code for computing the Jaccard similarity score and the Jaccard distance, together with an explanation of how it is applied to evaluate the similarity and dissimilarity between two causal graphs generated by our modified FCI algorithm. Additionally, a graphical representation illustrating the computation of the Jaccard similarity score and Jaccard distance is shown in Figure 3. In Figure 3a,b, two causal graphs to be compared are shown. Upon examining these graphs, it can be observed that only two edges are common, by taking into account the direction or type of the edge. These common or intersecting edges are depicted in Figure 3c, while the union of the two causal graphs (a) and (b) is illustrated in Figure 3d. According to [62], the Jaccard similarity score is calculated as the ratio of the length of the intersection graph (i.e., 2) to the length of the union graph (i.e., 6). And Jaccard distance, which measures the dissimilarity between the two graphs, is just a complement of the Jaccard similarity score. Therefore, Jaccardsimilarityscore=2/6=0.33, and Jaccarddistance=1−Jaccardsimilarityscore=0.67.

By tracking fluctuations in the Jaccard distance between causal graphs over preceding time periods compared to the reference graph, domain experts can detect deviations from the optimal operational state. They discern significant deviations using a predefined threshold value, established based on their process knowledge. When the Jaccard distance exceeds this threshold, experts investigate process dynamics changes using the causal graph. Analyzing the causal graph associated with notable Jaccard distance shifts enables experts to identify alterations and comprehend their consequences. This approach empowers domain experts to take informed corrective actions to restore the production process to normal conditions. By making process dynamics understandable, this method offers valuable insights, enabling proactive decision-making.
**Algorithm 1** Jaccard similarity and Jaccard distance calculation [62] 1:**Function** $\mathrm{JaccardSimilarity}(S_{1},S_{2})$ 2:   $\mathrm{intersection}\leftarrow |S_{1}\cap S_{2}|$ 3:   $\mathrm{union}\leftarrow |S_{1}\cup S_{2}|$ 4:   **return** $\frac{\mathrm{intersection}}{\mathrm{union}}$ 5:  6:**Function** $\mathrm{JaccardDistance}(S_{1},S_{2})$ 7:   **return** $1-\mathrm{JaccardSimilarity}(S_{1},S_{2})$ 8:  9:**Function** CalculateSimilarityAndDistance(graph1,graph2)10:   $S_{1}\leftarrow \left\{\mathrm{types}\;\mathrm{of}\;\mathrm{edges}\;\mathrm{between}\;\mathrm{the}\;\mathrm{node}\;\mathrm{pair}\;\mathrm{in}\;\mathrm{graph}1\right\}$11:   $S_{2}\leftarrow \left\{\mathrm{types}\;\mathrm{of}\;\mathrm{edges}\;\mathrm{between}\;\mathrm{the}\;\mathrm{node}\;\mathrm{pair}\;\mathrm{in}\;\mathrm{graph}2\right\}$12:   $\mathrm{similarity}\leftarrow \mathrm{JaccardSimilarity}(S_{1},S_{2})$13:   $\mathrm{distance}\leftarrow \mathrm{JaccardDistance}(S_{1},S_{2})$14:   **return** similarity,distance

## 4. Case Study

### 4.1. Process Description

**Figure 4:**
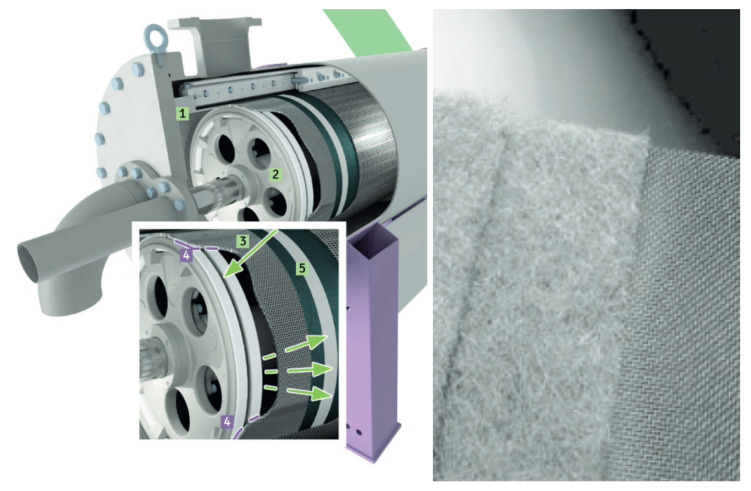
The filtration machine (**left**) uses dedicated metal sieves (**right**) to filter particles out of fluid viscose. Source: Lenzing ViscoFil (https://www.lenzing-technik.com/fileadmin/template/images/content/produkte/ViscoFil/ViscoFil_01-23_EN.pdf (accessed on 2 June 2024)).

The objective of this study is to investigate the applicability of causal discovery methods to monitor and detect deterioration in viscose fiber production. While this procedure consists of multiple steps, we focus on the most crucial one in terms of quality outcomes, the removal of particles in fluid viscose by filtration. Dedicated machinery is used for the filtration; see Figure 4. Due to the natural base of the viscose fluid and the molecular chaining that goes with it, particles can accumulate on the sieve used for the filtration. Thus, the filtration process outcome deteriorates and the filter requires regular washing, i.e., by a process called rejection. Eventually, the sieve needs to be replaced after continuous usage for several months to ensure high quality outcomes. Note that an individual filtration machine is just a single part of a parallel operation, because even though individual machines may deteriorate or even malfunction, the overall operation needs to be working, so that the following production steps can operate continuously.

**Filtration Machine: Filtration and Rejection Phase**

**Figure 5:**
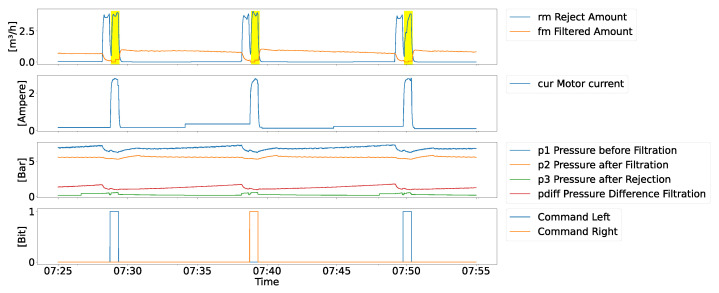
Exemplified data produced by the filtration machine. Pronounced peaks belong to the rejection phase (yellow highlight), the rest is part of the filtration phase.

In order to conduct filtration, the dedicated machinery (see Figure 4) operates on a mechanical principle; the fluid viscose is pushed by the pressure of the fluid through the sieve. Unwanted particles will not pass through the fine-grained sieve while fluid viscose will pass. As the filtered particles will block the sieve over time, the machine includes a back-washing operation mode, essentially reversing filtration direction and thus removing particles attached to the sieve and disposing them in a dedicated pipe system. Note that most of the time the machine operates in filtration mode, i.e., the filtration phase; however, based on reaching a dedicated differential pressure or the passing of time, the system triggers the rejection mode, i.e., the rejection phase. The rejection unit (2) moves from one side of the machine to the other. Along the crack within the rejection unit (3) the machine reverses the fluid direction sucking material in, which is opposed to the filtration direction which is pushing fluid from inside out. Note that a dedicated seal (4) separates the filtration and rejection operations, in order to avoid high amounts of material being wasted. As seen in Figure 5, the two phases produce very different shapes of signals and are thus independently analyzed in our causal discovery approach.

### 4.2. Data Description

The data we used for our analysis contain the multivariate time series depicted in Figure 5 and described in Table 1. In includes high frequency data (i.e., fmFilteredAmount at 11.76 Hz) but also very low frequency data like the control signals for the process (i.e., CommandRight at 0.0012 Hz). The data were thus resampled to 10 Hz either by interpolation (i.e., all values in Figure 5) or forward fill (command signals like start and stop of rejection process).

In Table 1, the range of sensor values along with the median value instead of the mean is presented, as sensor values are strongly affected by filtration and rejection processes. In general, the operation principle of the process is to keep the pressure values and the amount filtered rather stable; see Figure 5 and within the boundaries. For pressure, this is to avoid damage to the machine or any other component used. For the filtered amount, this is to keep downstream tasks continuously operating. Note that during the rejection phase, i.e., where either the machine moves the reject unit from left to right or right to left (as seen by CommandRight in Figure 5), pronounced peeks in motor current draw (cur) or reject amount (rm) are visible and the filtered amount (fm) drop slightly. Naturally, also the pressure values are slightly affected during and slightly after the rejection phase, including a small dent in p1, p2, and pressure difference and a small rise in p3 (reject pressure). The typical rejection phase is 40 s in length but can be delayed if the reject unit moves slower then anticipated, e.g., because of mechanical factors, like the condition of the motor. In Figure 5, during filtration due to more and more material blocking the sieve, the p1 pressure rises slightly, requiring a rejection phase to be within optimal operating conditions. As already mentioned, the sieve deteriorates due to the chemical and mechanical process, and thus the impact of the rejection phase also deteriorates. That in turn requires that the sieve inside the machine is changed regularly after several months of continuous usage. Therefore, the analysis considers a whole sieve life-cycle, i.e., it starts right after the sieve is changed for 5 months afterwards, i.e., where they are typically changed again.

### 4.3. Degradation Monitoring

This section provides an overview of the methodology and approach utilized to monitor degradation in the viscose fiber production process as described in Section 4.2. Figure 1 illustrates the approach employed for monitoring the degradation of the process over time.

As previously discussed in Section 4.1, the production process comprises two distinct phases: the filtration and the rejection phases. The duration of the rejection cycle remained constant at 40 s for each filter group, which was used for further analysis. However, the duration of the filtration cycle exhibited variability based on factors such as the sieve’s condition (whether it was new or old), amount of material blocking the sieve, the differential pressure, etc., as already discussed in Section 4.1. To accommodate this variability, the average duration of the filtration cycle over one month following a sieve change was computed. The calculated average duration was found to be 5.17min, which was used for further analysis. This information was needed for incorporating lags per feature as additional features within the dataset to obtain the causal graphs and is described below.

**Figure 6:**
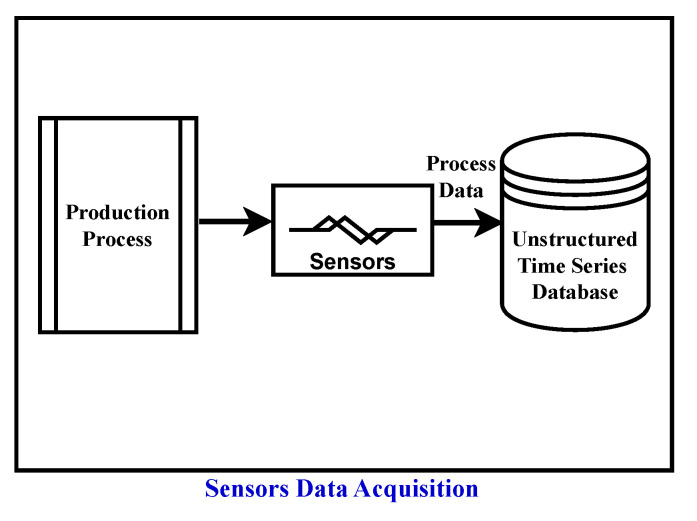
Sensor data acquisition stage.

**Figure 7:**
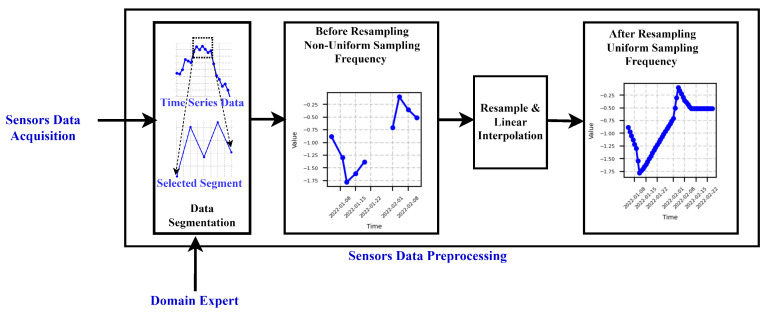
Sensor data preprocessing stage.

**Sensor Data Preprocessing:** The data obtained from the sensors, depicted in Figure 6, undergo prerocessing steps, as visualized in Figure 7. Firstly, the dataset was divided into two phases based on the respective times of filtration and rejection, as shown in the Data Segmentation part of Figure 7.

To address the irregular sampling frequency inherent in the rejection and filtration phases, we used data resampling techniques, as depicted in Figure 7. Specifically, the rejection phase data underwent resampling at a rate of 1 s, while the filtration phase data were resampled at a rate of 7 s. These resampling rates were determined based on recommendations from domain experts, ensuring alignment with the desired precision level for the analysis, particularly concerning the dynamic behavior of the process. This selection reflects the understanding that the dynamics of the process in the rejection phase exhibit faster variations compared to those in the filtration phase. For the sake of readability, here we focus on the rejection phase and the results for the filtration phase can be found in Appendix A.

**Figure 8:**
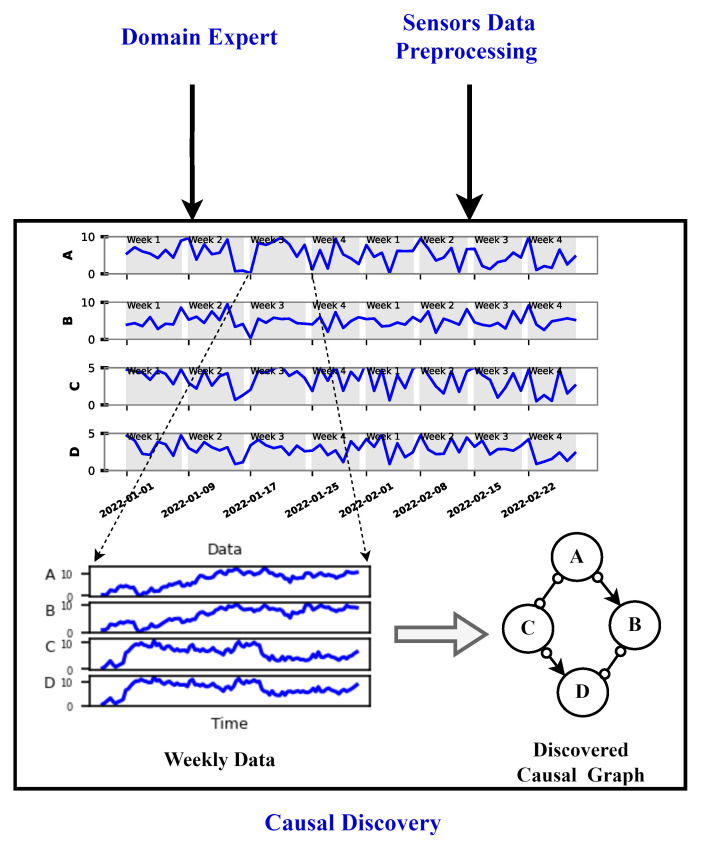
Causal discovery stage, where *A*, *B*, *C* and *D* are the nodes representing features/variables of the process.

**Causal Discovery:** At a frequency of 1 s, the rejection group data were obtained after completing the preprocessing step. Subsequently, these data were partitioned on a monthly basis, further dividing each month into four distinct weeks as shown in Figure 8. This segmentation strategy was implemented to facilitate the monitoring of degradation in the viscose fiber production process on a weekly basis. The decision to operate on a weekly frequency was motivated by the computational cost and time-consuming nature of causal graph computation. The computation complexity of the causal graphs using FCI is discussed below. Daily monitoring was deemed impractical, while monthly intervals were considered too infrequent, risking potential losses in the efficiency of the entire viscose fiber production system. As a result, the weekly basis provided a balanced and effective approach for a timely degradation assessment.

To monitor deterioration, the dataset described in Section 4.2 and Table 1, comprising seven features, was utilized. Furthermore, 40 lags per feature were included as additional features, where a lag represents the time delay between consecutive observations, indicating the temporal relationship between a variable and its past values as described in Section 3.2. To adapt FCI for time series data, additional features in the form of lags were introduced as described in Section 3.2. These lags serve as supplementary variables, facilitating the integration of temporal information into the causal discovery process. This modification allows FCI to account for the temporal dependencies present in time series data and uncover causal relationships that extend across different time points. The choice of the number of lags was influenced by the total duration of the rejection phase (40 s), along with its respective sampling frequencies (1 s), to ensure coverage of the entire duration of the rejection phase in the construction of the corresponding causal graphs. To ensure comparability between results for the rejection and filtration phases, domain experts recommended using the same number of lags for both phases. Consequently, by considering a total lag of 40 and a sampling frequency of 7 s, we covered almost the entire duration of the filtration phase (approximately 4.78min) in constructing the corresponding causal graphs. This harmonization of lag features enables consistent analysis across both phases of the production process.

Therefore, the total number of features required to construct causal graphs for both the rejection and filtration phases amounted to 7+7×40=287 features for each time point. With such a large number of features (287) per causal graph, and also considering the computational complexity of FCI, only two days of data were considered to represent the entire week. Each week, we used data from the first two successive complete days to create causal graphs for both the rejection and filtration phases. This resulted in around 19,000 samples with 287 features each. Constructing these graphs with FCI took approximately 6 h. During the creation of causal graphs using the FCI causal discovery method, domain knowledge emphasizing the principle that future or present events cannot influence past events was incorporated. This integration ensured that the causal graphs accurately reflected the causal relationships inherent in the dynamic production process.

**Figure 9:**
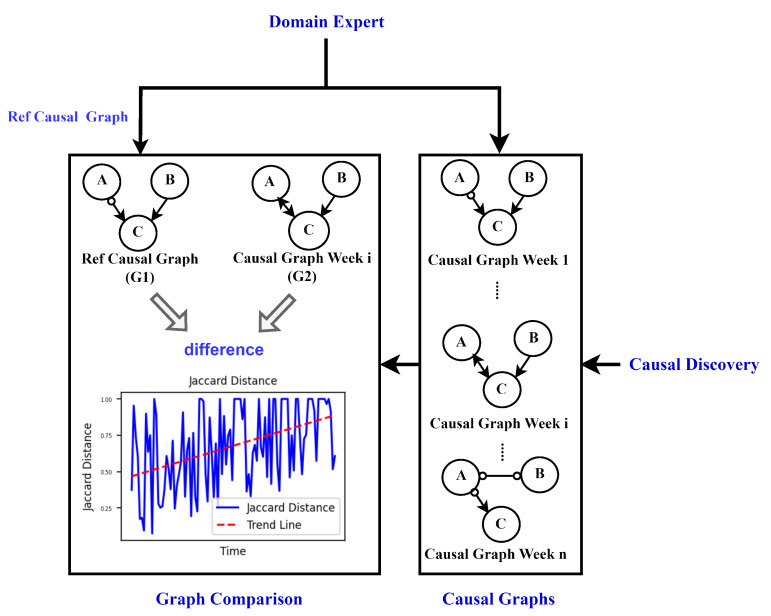
Causal graphs and graph comparison stages, where *A*, *B* and *C* are nodes representing features/variables of the process.

**Causal Graphs and Reference Causal Graph:** With the approach mentioned above, a total of 19 causal graphs were generated, each representing a specific week of each month from August (after the sieve was changed) to December 2022 as shown in the Causal Graphs Stage in Figure 9.

To effectively monitor the degradation of the process over time, a reference graph was pivotal. This reference graph would represent the normal operating scenario when the system functions as expected by the domain experts. The selection of such a reference graph is crucial for an accurate comparison of the graphs generated for consecutive weeks.

The criteria for choosing the reference graph involved selecting a graph that is close to the date when the sieve was changed and exhibits similarity to other causal graphs for the remaining weeks and months. The similarity between the graphs was quantified using the Jaccard similarity explained in Section 3.3, where a score of 0 indicates complete dissimilarity, and a score of 1 signifies identical graphs. The Jaccard similarity score was calculated while considering the direction of the edges between features, as FCI generates different types of edges as shown in Figure 2.

**Figure 10:**
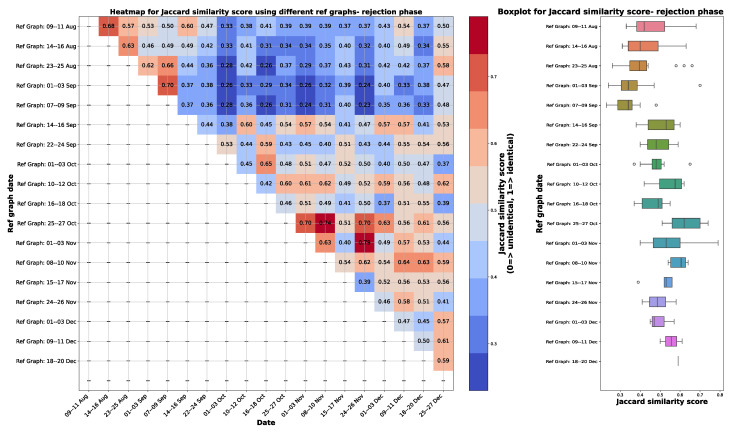
The heatmap on the left illustrates Jaccard similarity scores for various combinations of causal graphs used as reference graphs during the rejection phase. On the right, accompanying boxplots display the distribution of Jaccard similarity scores when a specific graph is selected as the reference and compared to the other causal graphs, where “°” indicates outliers in the data. The highlighted purple boxplot represents the chosen causal graph used as the ultimate reference, situated on 9–11 August 2022. This boxplot exhibits the highest median and is closest to the date of the sieve change, making it the selected reference graph for further analysis.

A heatmap depicting the Jaccard similarity score for different combinations of reference graphs during the rejection phase is presented on the left-hand side of Figure 10. This figure illustrates the computation of Jaccard similarity scores for various combinations of graphs used as reference graphs. The iterative process entails selecting one graph from all causal graphs as a reference and evaluating its similarity against all other graphs to identify the one exhibiting the highest resemblance to the others. In particular, this comparison excludes self-referencing (i.e., a graph is not compared against itself), and comparisons with graphs occurring before the reference are excluded to focus solely on monitoring degradation from the optimal state. Consequently, the heatmap is configured with only n∗(n−1)/2 entries, where n=19, corresponding to the total number of causal graphs.

On the right side of Figure 10, boxplots depict the distribution of Jaccard similarity scores when individual graphs are considered as the reference and compared with others. The choice of the reference graph aims to find one close to the date of the sieve change with a higher median and lower variance in Jaccard similarity scores, as shown in the right-hand side of Figure 10. This selection process is crucial as the reference graph should represent the ideal operating condition and be highly similar to other graphs, given that degradation is a gradual process. A higher median ensures greater similarity between the reference graph and others, reflecting the desired operational state. Meanwhile, lower variance indicates less significant variation among graphs, aligning with the gradual nature of degradation.

Among the examined boxplots, the graph depicting 9–11 August 2022, highlighted in purple, demonstrates the highest median and proximity to the sieve change date. Although the graph of 14–16 August 2022 also aligns closely with the sieve change date and exhibits similar variance in Jaccard similarity scores, it possesses a lower median compared to the one of 9–11 August. Consequently, the graph of 9–11 August was selected as the reference for further analysis. This decision ensures that the chosen reference graph effectively captures the optimal operating condition while maintaining consistency with the observed data dynamics.

**Figure 11:**
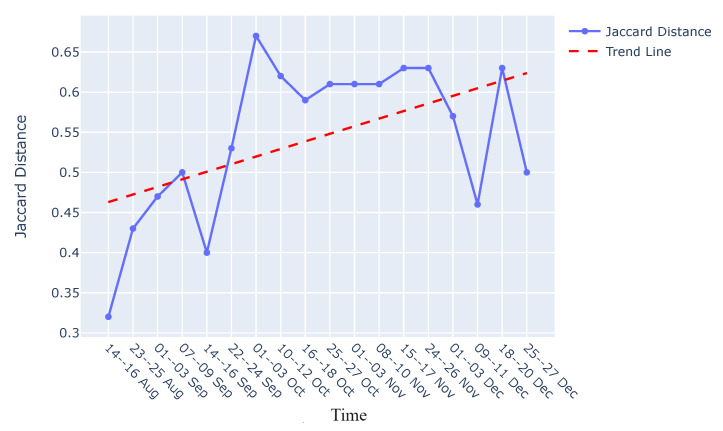
Jaccard distance for graphs obtained by using data from 2 days/week when compared to a graph on 9–11 August during the rejection phase.

**Graph comparison:** Once the reference graph was chosen, a comparative analysis was conducted with graphs over preceding time intervals using Jaccard distance, as illustrated in the graph comparison stage in Figure 9. The selection of the Jaccard distance as the comparison measure, instead of the Jaccard similarity score, was driven by the need to quantify the differences in causal graphs over time, as detailed in Section 3.3. These differences in causal graphs stem from variations in the dynamics of the sieve due to its degradation or deterioration during its operational span. Figure 11 visually presents the comparison between causal graphs and the reference graph (chosen to be the one on 9–11th August) using Jaccard distance for the rejection phase. Given the dynamic nature of the process, susceptible to variations over time, a trend analysis was performed after computing the Jaccard difference score to monitor degradation in the production process. The observed positive trend indicates an increase in degradation over time following the change in the sieve.

**Figure 12:**
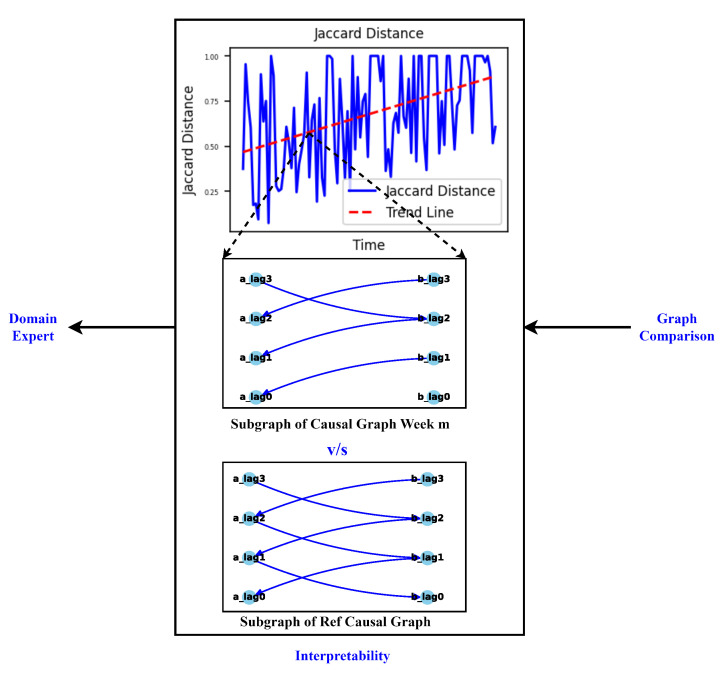
Interpretability stage.

**Interpretability:** Our approach not only facilitates the continuous monitoring of degradation in the viscose fiber production process but also empowers domain experts to integrate their knowledge into the creation and interpretation of causal graphs. As shown in Figure 12, this section focuses on interpreting the observed variations in the dynamics of the production process during degradation monitoring, employing two distinct methods.
*Visual Inspection of Causal Graphs for Root Cause Analysis:* The initial method involves visually examining causal graphs to discern changes at specific time points. By setting a degradation threshold for the Jaccard distance, as demonstrated in Figure 11, domain experts can scrutinize changes and analyze the causal graph of the ongoing production process.

**Figure 13:**
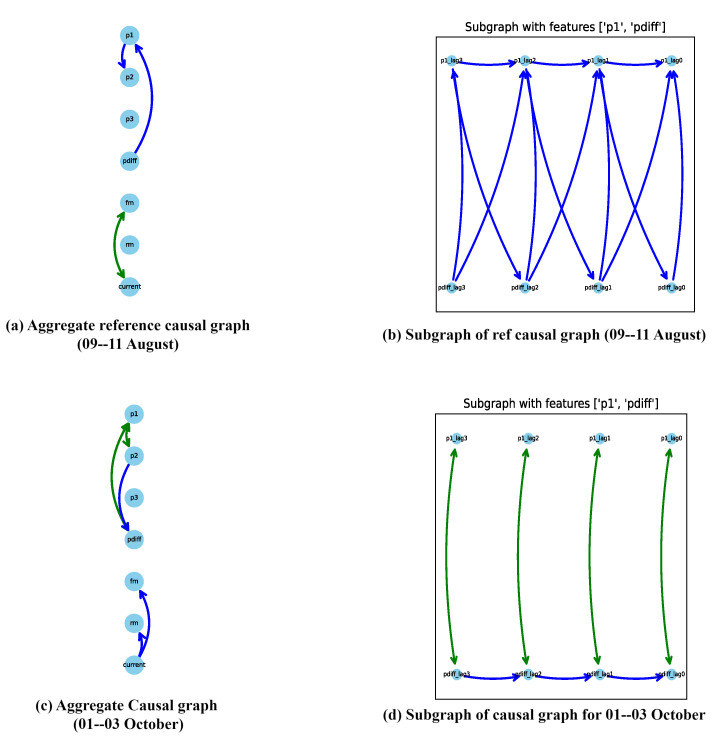
Extraction of causal graphs for 9–11 August and 1–3 October. (**a**) Simplified reference causal graph (9–11 August). (**b**) Subgraph of the reference graph with features *p*1 and *pdiff*. (**c**) Simplified causal graph for 1–3 October. (**d**) Subgraph of causal graph for 1–3 October.

For example, considering the maximum Jaccard distance on 1–3 October from Figure 11, a comparison between the causal graphs for the reference graph (9–11 August) and this date (1–3 October) is performed. Figure 13a,c showcase the aggregate causal graphs for the reference graph (9–11 August) and 1–3 October, respectively. The complete causal graph is inherently dense, featuring 40 lags per feature. Due to the repetition of edges between feature pairs over time, the simplified causal graph is presented to emphasize connections between features over a single lag. The edge connectivity between feature pairs or nodes repeats as the graph unfolds in time, and thus, only the unique patterns are illustrated in Figure 13a,c.

Upon thorough analysis, several notable changes emerge, particularly evident in the causal graph on 1–3 October depicted in Figure 13c compared to the reference graph on 9–11 August shown in Figure 13a. One significant observation is the introduction of latent confounders in the causal graph on 1–3 October, which are absent in the reference graph. An in-depth examination of the subset graph for both dates, focusing on features *p*1 and *pdiff* in Figure 13b,d, reveals the emergence of a latent confounder influencing their relationship in the causal graph on 1–3 October, whereas it was absent in the reference causal graph on 9–11 August. This relationship holds crucial significance as it triggers the initiation of the rejection and filtration phases, making the introduction of a latent confounder a critical observation.

The differential pressure (*pdiff*) signifies the disparity between the input pressure (*p*1) and the constant output pressure (*p*2). Thus, variations in *p*1 directly impact *pdiff*, given the constant nature of *p*2. When *pdiff* exceeds a certain threshold, rejection initiates; otherwise, filtration continues. However, the introduction of a latent confounder enables false switching between the rejection and filtration phases, impacting output quality in multiple ways. Firstly, an increased number of filtrations and fewer rejections may indicate insufficient space within the sieve for new waste particles, leading to clogging and reducing the lifespan of the sieve and degrading the output quality. Alternatively, excessive rejections may result in more frequent motor contact with the sieve during cleaning or backwashing, accelerating mechanical degradation and shortening the sieve’s lifespan, subsequently diminishing output quality. This observation underscores the importance of identifying and addressing latent confounders to maintain process integrity and ensure optimal output quality.

Further examination shows a delayed connection between *p*1 and *pdiff* in the reference graph (Figure 13b) that is absent in the causal graph for 1–3 October (Figure 13d). Visual inspection thus provides domain experts with valuable insights into changes in feature relationships, thereby providing a basis or an initial point for the future analysis.

The latent confounders not only exist between features *p*1 and *pdiff* but also extend to include the features *p*1 and *p*2. Additionally, new connections emerge in the causal graph for 1–3 October, as depicted in Figure 13c, which are not present in the reference causal graph shown in Figure 13a. This comprehensive analysis provides domain experts with a more profound insight into the evolving dynamics of the process.

*Monitoring Changes in Feature Relations Over Time:* The second approach involves monitoring changes in the relationship between specific pairs of desired features over time. As previously mentioned, the connections between features *p*1 and *pdiff* play a crucial role in initiating the rejection and filtration phases. Therefore, observing the dynamics of these features over time can provide valuable insights before a significant event occurs.

**Figure 14:**
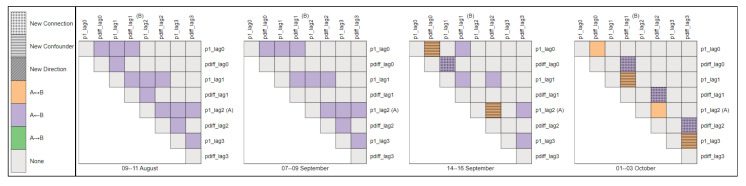
Visualization of the changes in the relation between the features *p*1 and *pdiff* over time. Starting with week 11–16 September we see the introduction of additional confounders hinting to changes in the underlying process.

The proposed visualization in Figure 14 provides an insightful depiction of the monitoring process over time. Notably, between 9–11 August and 7–9 September, no confounders or latent variables are observed between features *p*1 and *pdiff*, as indicated in the corresponding heatmaps. However, a crucial development occurs on 14–16 September, highlighted in orange on the heatmaps, signaling the appearance of latent confounders in the causal graph. This identification empowers domain experts with the knowledge of when these confounders emerged, enabling focused root cause analysis during this timeframe to discern the underlying causes of such occurrences. Armed with this information, experts can strategize how to maintain process dynamics to meet required specifications.

Moreover, the visualization serves to highlight any new connections or confounders compared to previous causal graphs. This functionality allows domain experts to swiftly detect irregularities in process dynamics while considering the ideal operating scenario derived from the reference causal graph. By leveraging this visualization, experts can proactively address deviations from optimal process conditions, ensuring consistent performance and quality output.

Upon examination of Figure 14, a noticeable trend emerges wherein potential confounders appear to proliferate over time. This trend underscores the significance of ongoing monitoring, enabling domain experts to discern abnormal behavior and initiate deeper investigations. By dynamically tracking these changes, valuable insights into the evolving relationships between features are gleaned, facilitating the early detection of anomalies or shifts in the production process dynamics. The inherent advantage of leveraging causal graphs lies in providing domain experts with targeted insights: from these heatmaps, experts discern which causal graph to scrutinize, subsequently gaining clarity on the underlying reasons for observed changes and informing their investigative focus to uphold output quality standards.

## 5. Evaluation

We conducted an offline evaluation to assess the effectiveness of our degradation monitoring method. In this evaluation, we utilized an LSTM-based Autoencoder, TranAD, and USAD, employing them to monitor degradation in the process.

### 5.1. LSTM Based Autoencoder

The idea behind using an LSTM-based Autoencoder (AE) for anomaly detection is to take advantage of the capability of LSTMs to capture temporal dependencies (since we are dealing with time series data). LSTMs, or Long Short-Term Memory networks, are a type of Recurrent Neural Network (RNN) that is well-suited for modeling sequences. In contrast to our method, the LSTM-based algorithm cannot be considered to be interpretable. The method follows the same principles as outlined by [21] and described in Section 2.2. However, a distinctive feature of our evaluation is the setting of the threshold to the 99 percentile of the mean absolute error of the training error, as elucidated in Section 5.1.1. The determination of this threshold was guided by insights from domain experts.

#### 5.1.1. Procedure and Results

During the training phase, the LSTM-based Autoencoder is exposed to sequences of data collected during normal operating conditions. The model learns to reconstruct or predict the input sequences. The autoencoder consists of an encoder with two LSTM layers that compress the input data into a latent/hidden space representation and a decoder that reconstructs the input data from this representation.

The LSTM-based autoencoder was trained on normal data, specifically the same dataset used to construct the reference graph described in Section 4.3, which corresponds to the data from 9–11 August. This training was designed to teach the model the typical patterns, structures, and dependencies present during regular operation, allowing it to capture the inherent regularities and variations in normal sequences. During training, 5% of the data was reserved for validation and the model underwent 100 training epochs. The dataset used for training consisted of approximately 19,000 data samples, with 950 samples reserved for validation. The remaining 18,050 samples were used for training.

The evaluation utilized test data comprising consecutive weeks from August 2022 to December 2022, excluding the data utilized for constructing the reference graph (9–11 August). This test dataset consisted of complete data for consecutive weeks, amounting to approximately 19,000 data samples. Using the identical dataset for both training and testing with the LSTM-based autoencoder, just as with our approach, ensured a fair comparison between the two methods for monitoring degradation.

**Figure 15:**
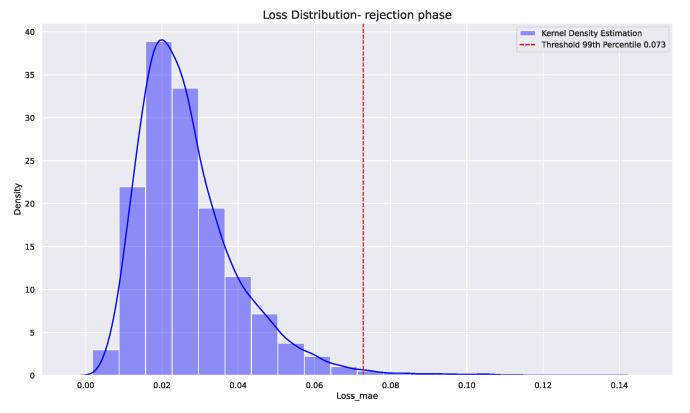
Distribution of the reconstruction loss (mean absolute error) using kernel density estimation on the training dataset with a threshold set at 0.073 which is 99 percentile of the mean absolute error for the rejection phase.

After training the model, anomalies are typically detected by setting a threshold on a reconstruction error metric. The reconstruction error is calculated by comparing the input sequence with its reconstructed version. In this case, the loss function used was the mean absolute error and the threshold was set to 99 percentile of the mean absolute error of the training error. This threshold was set based on the knowledge from the domain expert. Figure 15 shows the distribution of the reconstruction loss of the model over the training dataset with 99 percentile set as a threshold for anomalies in the test data. The kernel density distribution of the reconstruction loss is computed. When the reconstruction error on the test data surpasses this threshold, it indicates an anomaly.

**Figure 16:**
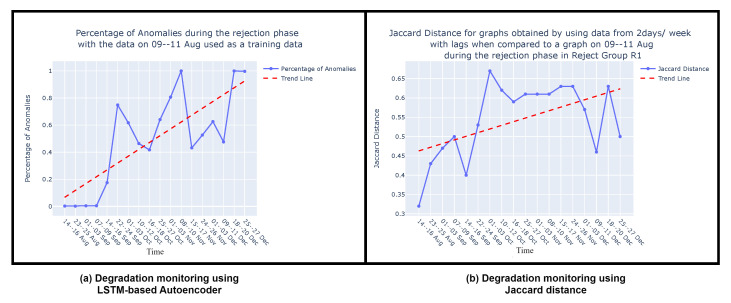
Degradation monitoring for rejection phase using (**a**) LSTM-based Autoencoder and (**b**) Jaccard distance of our proposed approach.

Once the model is trained, it can be applied during the testing or deployment phase to predict or reconstruct new sequences. Anomalies or deviations from normal patterns are identified when the model struggles to accurately reconstruct or predict the input sequence. In Figure 16a, the percentage of anomalies in the total test data during the rejection phase is depicted, along with a trend analysis. As discussed in Section 4.3, the dynamic nature of the process, which is susceptible to variations over time, leads to fluctuations in the percentage of anomalies, which is similar to the variability observed in the Jaccard distance (Section 4.3, Figure 16b). Consequently, a trend analysis for the LSTM-based AE approach was also conducted to discern the percentage of anomalies over time. The trend line reveals an increasing percentage of anomalies from August to December, indicating degradation in the sieve. These findings corroborate the results discussed in Section 4.3 and are visually depicted in Figure 16b. It is important to note that this comparison focuses solely on the shape of the trend lines—whether they vary positively or negatively—and does not involve comparing the slope of the trends. Both methods demonstrate the ability to monitor degradation in the viscose fiber production process, as is evident in Figure 16a,b.

### 5.2. TranAD and USAD

The TranAD method stands out as an advanced approach for monitoring degradation in the viscose fiber production process. It takes advantage of deep transformer network, offering a sophisticated framework for anomaly detection and diagnosis. Unlike conventional methods, TranAD incorporates attention-based sequence encoders, enabling it to analyze data efficiently while capturing broader temporal trends effectively. Moreover, it utilizes focus score-based self-conditioning to extract robust multi-modal features, ensuring a comprehensive understanding of the data dynamics. In addition, the model employs adversarial training techniques to enhance stability and resilience against noise and perturbations [29].

The architecture of TranAD resembles other transformer-based models, featuring an encoder–decoder structure. Specifically, the encoder processes the entire sequence up to the current timestamp, utilizing focus scores to weigh the significance of each data point. Subsequently, a window encoder aggregates this information to create an encoded representation of the input window. This representation is then fed into two decoders to reconstruct the original sequence. For a more in-depth understanding of the workings of TranAD, interested readers are encouraged to explore the detailed insights provided in [29].

The UnSupervised Anomaly Detection (USAD) method operates on an autoencoder (AE) architecture, drawing inspiration from Generative Adversarial Networks (GANs) to enhance its learning capabilities. USAD’s core principle involves training its encoder–decoder architecture to amplify the reconstruction error of inputs containing anomalies, while ensuring stability, which is a common challenge in GAN-based approaches. Unlike traditional autoencoders, USAD leverages adversarial training to address issues such as mode collapse and non-convergence, which are commonly encountered in GAN architectures.

The USAD model consists of an encoder network *E* and two decoder networks D1 and D2. These components form an architecture comprising two autoencoders AE1 and AE2, both sharing the same encoder network. Mathematically, the formulation is as follows [32]:

AE1(W)=D1(E(W)),AE2(W)=D2(E(W))

Training USAD involves two phases. Initially, the two autoencoders are trained to reconstruct normal input windows *W*. Subsequently, they undergo adversarial training, where AE1 attempts to deceive AE2, while AE2 learns to distinguish between real data (directly from input window *W*) and reconstructed data (from AE1). This two-phase approach enables USAD to effectively identify anomalies while maintaining stability during training [32].

However, while these methods can effectively monitor degradation in the viscose fiber production process, our approach offers an additional advantage of transparency. This is achieved through the comparison of causal graphs over time, enabling us to discern changes that occurred throughout the monitoring period. Considering that the assessment protocol for degradation monitoring using TranAD and USAD mirrors the same steps and relies on the identical dataset, the procedure and results for both techniques are addressed collectively Section 5.2.1.

#### 5.2.1. Procedure and Results

The data used for training TranAD and USAD consisted of normal data, similar to the approach used for LSTM-based Autoencoder, and the data used to construct the reference causal graph, specifically from 9th August to 11th August. To capture temporal dependencies, both TranAD and USAD utilized windows, with a window length set to 40 to match the number of lags considered in creating the causal graph as described in Section 4.3.

Prior to training, the data underwent scaling using the min-max scaler, and the time column was excluded. This was because temporal information is inherently encoded within the window in the operational process of both models, namely TranAD and USAD. Both TranAD and USAD were trained on a dataset consisting of 19,000 data samples, which were the same as those used for constructing the reference causal graph, as described in Section 4.3. Once trained on normal data, the models were tested on subsequent week’s data, excluding the data used for training. The test dataset also comprised 19,000 data samples consistent with the data used in creating causal graphs for subsequent weeks.

Anomalies were identified by applying a threshold established during training, which was determined by the contamination parameter representing the percentage of outliers in the training data. For this study, the contamination parameter was set to 0.1 based on domain knowledge of the viscose fiber production process.

Following the training and testing phases, the models’ performance was evaluated by computing the percentage of anomalies detected in the test data, representing the subsequent week’s data. Subsequently, a trend analysis was conducted to discern degradation trends within the viscose fiber production process.

**Figure 17:**
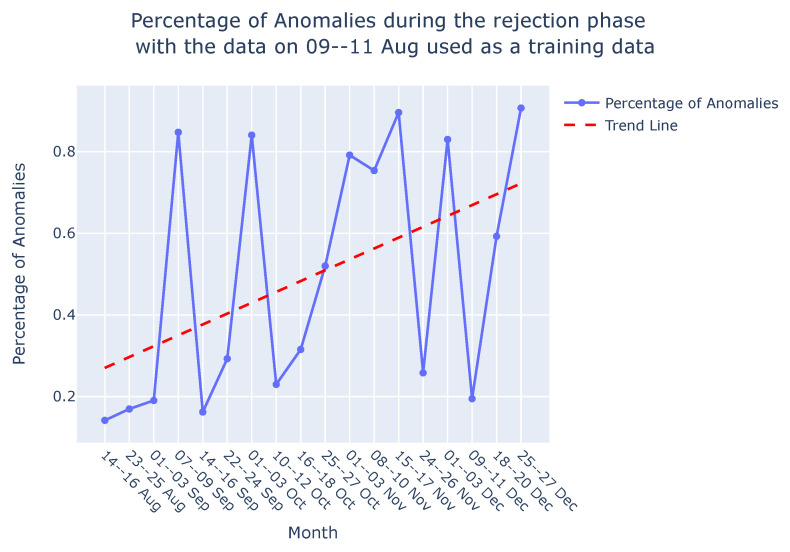
Degradation monitoring for rejection phase using TranAD.

**Figure 18:**
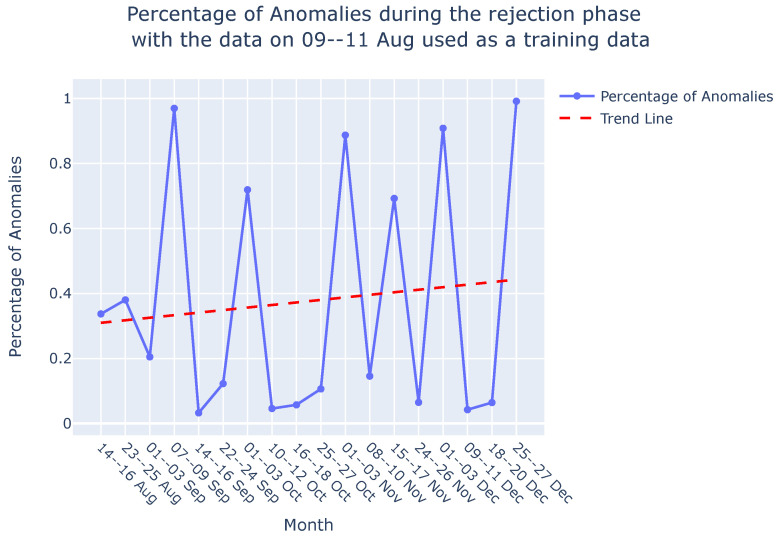
Degradation monitoring for rejection phase using USAD.

Figure 17 and Figure 18 illustrate the percentage of anomalies observed in the total test data during the rejection phase using TranAD and USAD.

As mentioned earlier in Section 4.3, the dynamic nature of the process results in variations over time, leading to fluctuations in the percentage of anomalies. This phenomenon is evident in the results obtained from the LSTM-based AE, TranAD, and USAD, as depicted in Figure 16a, Figure 17 and Figure 18, along with the Jaccard distance displayed in Figure 16b.

In comparing our method with LSTM-based AE, TranAD, and USAD, our focus lies primarily on evaluating the overall trend shape rather than directly comparing specific degradation rates or anomaly percentages. While all the evaluation methods, including LSTM-based AE, TranAD, and USAD, aim to detect the percentage of anomalies over time, we analyze deviations in process behavior by comparing causal graphs using the Jaccard distance. This variance in approach makes direct comparison challenging. Nonetheless, all methods demonstrate a positive trend, suggesting increased degradation over time in the viscose fiber production process.

## 6. Conclusions and Future Scope

In conclusion, the application of degradation monitoring in the context of a highly dynamic production process, exemplified by viscose fiber production in our case study, has demonstrated to be a valuable strategy to maintain operational efficiency and ensure product quality. The use of causal discovery methods has added a layer of interpretability to the monitoring process, allowing domain experts to incorporate background knowledge and investigate changes in causal relationships over time.

While mechanistic models may struggle to capture the complexity of real-time industrial processes, data-driven methods offer a viable alternative. However, to facilitate effective decision-making, domain-specific evaluations are indispensable, as demonstrated in our causality analysis, where domain knowledge was added as a background knowledge in the creation of causal graphs. Unlike traditional black-box models, which provide limited insights, Explainable AI (XAI) tools like SHAP offer explanations for predictions [63]. Although SHAP is a popular choice, it assumes independence between features [64], which may not hold true in interconnected systems like the viscose fiber production process depicted in our data (Figure 5).

In contrast, our causality analysis offers a transparent white-box model, where causal relations between components are explicitly known. This enables the attribution of changes to degradation events rather than arbitrary shifts. Furthermore, by comparing causal graphs, our approach unveils alterations in relations over time, empowering domain experts to pinpoint critical deviations. Therefore, while SHAP and similar XAI tools provide valuable insights, their applicability may be limited in complex, interconnected systems where the assumption of feature independence does not hold. In such cases, causality analysis offers a more transparent and domain-aware approach to monitoring and understanding dynamic processes. The two-fold advantage of causal discovery, enabling the integration of expert knowledge and facilitating interpretable changes in causal relationships, has enabled domain experts to not only monitor degradation, but also delve into the root causes of variations. This, in turn, forms the basis for further investigations, including root cause analysis and counterfactual reasoning. In summary, the integration of causal discovery with Jaccard distance in degradation monitoring provides a pathway towards proactive maintenance, improved process stability, and a deeper understanding of the dynamic interplay of factors influencing production quality and efficiency.

In future work, understanding the specific causes of degradation becomes crucial, necessitating root cause analysis to pinpoint and replace defective components. Integrating this with conditional monitoring, where predefined criteria (e.g., Jaccard distance in our approach) exceeding a threshold trigger root cause analysis, ensures timely intervention to enhance fiber quality. Mechanical degradation, inherent in the aging of components such as sensors and sieves, underscores the importance of counterfactual reasoning. This approach provides insights into how alterations in specific variables might influence outcomes under varying conditions. This understanding facilitates proactive decision making and risk mitigation, ultimately improving fiber quality.

## Figures and Tables

**Table 1:** Information about the sensors used.

Abbrv.	Description	Min	Max	$\tilde{x}$	Unit	Avg. Sampling Period
p1	p1-Pressure measured before the machine	0.61	9.90	6.79	Bar	85 ms
p2	p2-Pressure measured after the filtration	0.00	6.36	5.54	Bar	1 s 60 ms
p3	p3-Pressure measured after the rejection	0.00	2.92	0.19	Bar	1 s 60 ms
pdiff	Pressure difference between before and after filtration	0.33	4.58	1.23	Bar	1 s 60 ms
fm	Amount of fluid that passed through the filter	0.00	8.55	0.68	m^3^/h	90 ms
rm	Amount of fluid which was rejected	0.00	10.0	0.02	m^3^/h	85 ms
current	Current used to move rejection unit motor	0.16	6.11	0.25	Ampere	1 s

**Figure A1:**
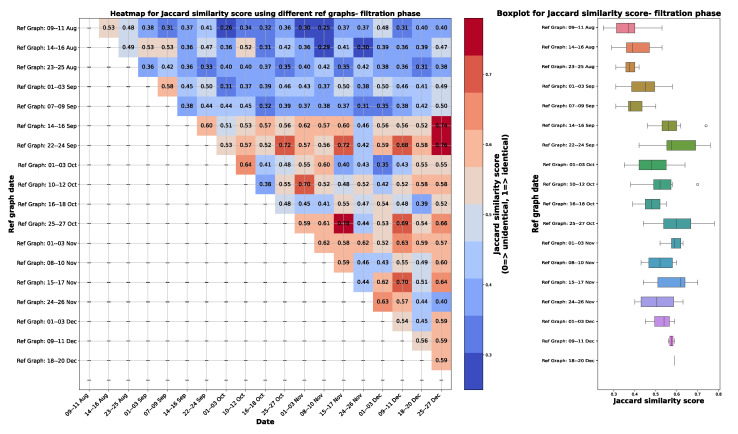
The heatmap on the left illustrates Jaccard similarity scores for various combinations of causal graphs used as reference graphs during the filtration phase. On the right, accompanying boxplots display the distribution of Jaccard similarity scores when a specific graph is selected as the reference and compared to other causal graphs, where “°” indicates outliers in the data. The highlighted purple boxplot represents the chosen causal graph used as the ultimate reference, situated on 9–11 August 2022. This boxplot exhibits the high median, low variance in the Jaccard similarity score, and is closest to the date of the sieve change, making it the selected reference graph for further analysis.

**Figure A2:**
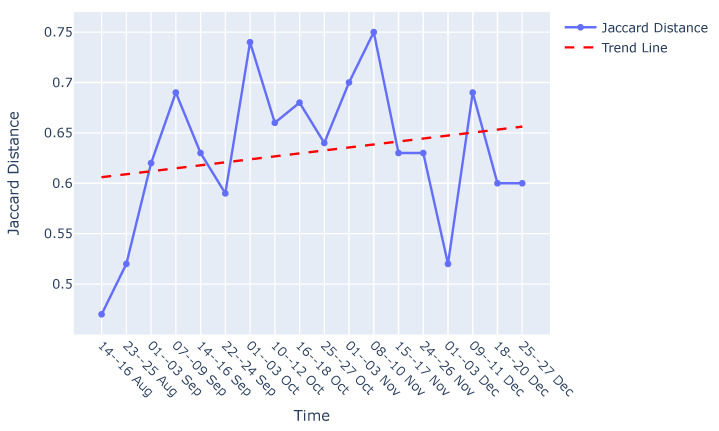
Jaccard distance for graphs obtained by using data from 2 days/week when compared to a graph on 9–11 August during the filtration phase.

**Figure A3:**
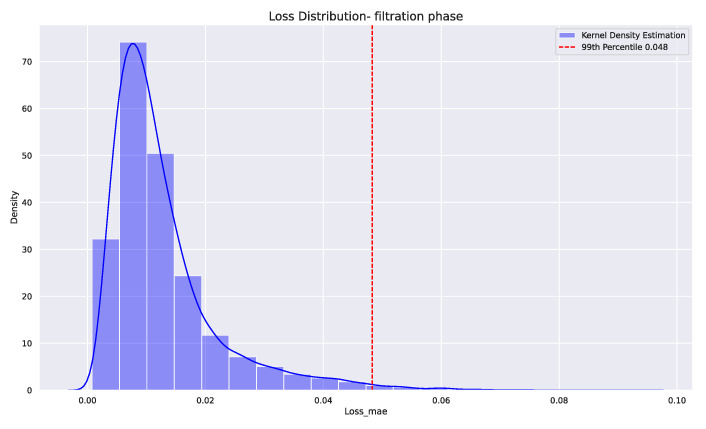
Distribution of the reconstruction loss (mean absolute error) using kernel density estimation on the training dataset with a threshold set at 0.078, which is the 99 percentile of the mean absolute error for the filtration phase.

**Figure A4:**
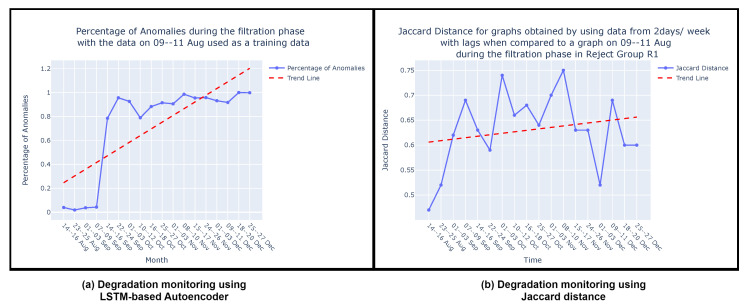
Degradation monitoring for filtration phase using (**a**) LSTM-based Autoencoder and (**b**) Jaccard distance.

**Figure A5:**
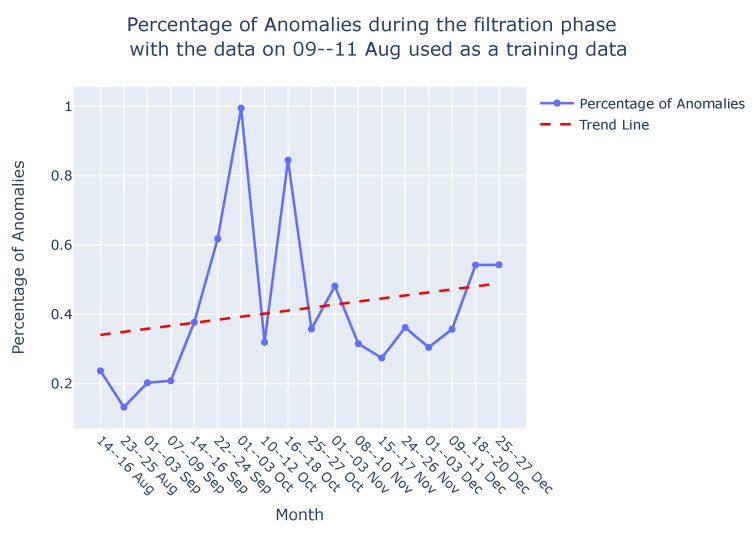
Degradation monitoring for filtration phase using TranAD.

**Figure A6:**
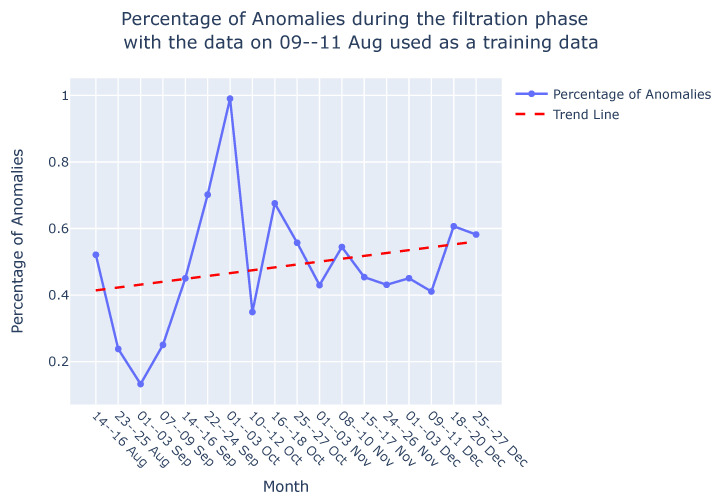
Degradation monitoring for filtration phase using USAD.

## Data Availability

Restrictions apply to the availability of these data. Data were obtained from Lenzing GmbH.

## Appendix A. Degradation Monitoring—Filtration Phase

This appendix presents the outcomes of monitoring the degradation in the filtration phase. It is important to note that the data utilized for the rejection and filtration phases correspond to the same dates, as outlined in Section 4.3. However, the data for the filtration and rejection phases has been segregated based on the respective times when filtration and rejection were active, as detailed in Section 4.3.

### Appendix A.1. Causal Graphs and Reference Causal Graph for Filtration Phase

As mentioned in Section 4.3, the criteria for selecting the reference graph involved identifying a graph close to the date when the sieve was changed and demonstrating similarity to other causal graphs in the subsequent weeks and months. The reference graph selection process focused on finding a graph near the date of the sieve change with a higher median and lower variance in the distribution of Jaccard similarity scores. The heatmap, displaying the Jaccard similarity scores for various combinations of reference graphs during the filtration phase, is presented on the left-hand side of Figure A1. The boxplots on the right-hand side of Figure A1 show the distribution of the Jaccard similarity scores when a specific graph is considered as the reference graph and compared with others. The graph on 9–11 August 2022, highlighted in purple, stands out with a high median, low variance, and proximity to the date of the sieve change. It is worth noting that the graph on 14–16 August 2022 could also be considered a reference, being close to the sieve change date with a high median, but it exhibits a higher variance in the Jaccard similarity score compared to the graph on 9–11 August. Therefore, the graph on 9–11 August was selected as the reference graph for further analysis.

### Appendix A.2. Graph Comparison

The visual representation in Figure A2 illustrates the comparison of causal graphs with the reference graph using Jaccard distance, incorporating a trend analysis for the filtration phase. The observed positive trend signifies a progressive increase in degradation over time subsequent to the sieve change.

## Appendix B. Evaluation—Filtration Phase

In this section, we showcase the outcomes of the offline evaluation conducted to assess our approach for monitoring degradation in the filtration phase. Figure A3 displays the distribution of the training loss using kernel density estimation over the reconstruction loss, where the threshold is established at the 99 percentile (i.e., 0.078) of the reconstruction loss, denoted as the mean absolute error.

In Figure A4a, the percentage of anomalies over the total test data in the filtration phase is depicted, along with a trend analysis. The analysis utilized the LSTM-based Autoencoder model trained on the reference data, i.e., on 9–11 August. The mean absolute error, representing the reconstruction error, was calculated using data from consecutive weeks spanning August 2022 to December 2022, as outlined in Section 5.1.1. The trend line illustrates a progressively increasing percentage of anomalies from August to December, indicating degradation in the sieve. These findings align with the results discussed in Appendix A.2 and are visually represented in Figure A4b.

Figure A5 and Figure A6 depict the percentage of anomalies observed over the total test data during the filtration phase, accompanied by a trend analysis utilizing TranAD and USAD. Both models (TranAD and USAD) were trained on the same dataset used for creating the reference causal graph, specifically from 9th August to 11th August. The trend line illustrates a progressively increasing percentage of anomalies from August to December, indicating degradation in the sieve. These findings are consistent with the results discussed in Appendix A.2 and are visually represented in Figure A4b.
